# Elements of Pathological Anatomy

**Published:** 1858-01

**Authors:** 


NORTH AMERICAN

### MEDICO-CHIRURGICAL REVIEW.

JANUARY, 1858.

### Analytical and Critical Reviews.

ART. I.—*Elements of Pathological Anatomy.* By S. D. GROSS, M.D. Professor of Surgery in Jefferson Med. College, and formerly Professor of Pathological Anatomy in the Medical Department of Cincinnati College. Third edition, modified and thoroughly revised. Illustrated by three hundred and forty-two engravings on wood. 8vo, pp. 771. Philad. 1857.

*Rudiments of Pathological Histology.* By CARL WEDL, M.D., etc. With one hundred and seventy-two illustrations on wood. Translated and edited by GEORGE BUSH, F.R.S. London: Printed by the Sydenham Society, 1855. pp. 637, 8vo.

*The Pathological Anatomy of the Human Body.* By JULIUS VOGEL, M.D., Professor of Clinical Medicine in the University of Giessen. Translated from the German, with additions, by GEORGE DAY, M.D. and L.M., Cantab. etc. etc. Illustrated by upwards of one hundred plain and colored engravings, vol. 1. Philadelphia: Lea & Blanchard, 1847. pp. 534, 8vo.*

*An Anatomical Description of the Diseases of the Organs of Circulation and Respiration.* By CHARLES EWALD HASSE, M.D., Professor of Pathology and Clinical Medicine at the University of Zurich, etc. etc. Translated and edited by W. E. SWAINE, M.D. London: Printed for the Sydenham Society, 1846. pp. 400, 8vo.†

---

* This work was first brought out by the Sydenham Society.

† Dr. Hasse's volume was republished by Lea & Blanchard.

*A Manual of Pathological Anatomy.* By CARL ROKITANSKY, M.D., Curator of the Imperial Pathological Museum, and Professor of Pathological Anatomy at the University of Vienna, etc. In four volumes. London: Printed for the Sydenham Society, 1849–54.*

*A Compendium of Human and Comparative Pathological Anatomy.* By ADOLPH WILHELM OTTO, M.D., Professor of Medicine in the University of Breslau. Translated from the German, with Additional Notes and References, by JOHN F. SOUTH, Lecturer on Anatomy at St. Thomas's Hospital. London, 1831, pp. 456.

*A Treatise on Pathological Anatomy.* By G. ANDRAL, M.D. Translated from the French by Drs. RICHARD TOWNSEND and WILLIAM WEST. Republished in two volumes. New York, 1832, pp. 424 and 507, 8vo.

*A Treatise on Pathological Anatomy.* By WILLIAM E. HORNER, Adjunct Professor of Anatomy in the University of Pennsylvania, etc. etc. Philadelphia, 1829, pp. 460.

*Histoire Anatomique des Inflammations.* Par A. N. GENDRIN, D.M., etc. etc. Paris, 1826, 2 vols. 8vo, pp. 719 and 645.

*Histoire des Phlegmasies ou Inflammations Chroniques Fondée sur des Nouvelles Observations de Clinique et d'Anatomie Pathologique.* Par F. J. V. BROUSSAIS, etc. etc. 3 vols. 8vo, 1826. pp. 386, 649, and 492.

*Essai sur l'Anatomie Pathologique en General, et sur les Transformations et Productions Organiques en Particulier.* Paris, 1816. 2 vols. 8vo, pp. 400 and 461. Par JEAN CRUVEILHIER.

*Anatomie Pathologique.* Par LAENNEC and BAYLE, in *Dictionnaire des Sciences Médicales.*

*Anatomie Pathologique.* Par CRUVEILHIER, in *Dictionnaire de Médecine et de Chirurgie Pratiques.*

WE pray our readers not to be alarmed at this array of the titles of works on Pathological Anatomy, as if it implied an intention on our part either to give a review of their contents or to attempt an elaborately critical disquisition on their respective merits. We shall not engage in a labor of this kind, although we shall be found to refer occasionally to their pages as we advance. Our design might be aided by adopting either of the courses just mentioned; but we hope to reach the end which we propose to ourselves by a shorter and an easier process. It is, to demonstrate

* The second volume of this work was issued the first, then followed the third and fourth. Volume first was published the last. The entire work has been republished by Blanchard & Lea, in two volumes.

the importance of pathological anatomy to medicine proper, regarding the latter both as a science and an art; and to impress on the minds of the members of the profession generally, and of teachers more particularly, the necessity of giving the subject more prominence in their clinical pursuits and public prelections than it has hitherto attained. Perhaps we shall the better succeed in gaining attention by stating, at the outset, our reason for introducing the titles of the several works which head this article. These works are part of the library of the writer; they were not collected after a definite plan, nor because of his ever having made, either as an author or lecturer, pathological anatomy a special study and pursuit; but, simply, because he believed them to be a necessary constituent part of medical literature, and, as such, requiring equal attention, and to be placed on the same line as works on the other more generally-admitted divisions of medicine. The list might, without much bibliographical lore, be greatly extended. In taking a retrospective course we should come to the name of the great Morgagni and his meritorious but less distinguished predecessor, Bonetus. In the descending series the array would be considerable, including the last authoritative treatise by Dr. Gross, noticed in the September number of this journal, but with a brevity quite incommensurate with its claims on our consideration. An English scholar will point, in this department, to a Baillie, while, perhaps, overlooking the claims on our gratitude of a richer contributor, though not under the same title and form, in the person of John Hunter, whose treatise on the *Blood, Inflammation, and Gunshot Wounds*, is a continued application of physiological and pathological anatomy to an explanation of the changes in morbid structures, and of the recuperative processes set up for their limitation or removal.

Were it not that pathological anatomy has been sometimes confounded with pathology—a part substituted for the whole—it would be scarcely necessary to define the object and limits of the branch now before us. We may say, then, that anatomy, as the science which teaches the structure of organic bodies, is divided into—1, normal or physiological; and 2, pathological or morbid, and, in reference to its application, into practical or medical anatomy. The last designation—medical—was applied by Harvey, whose physiological inquiries and great discovery did not prevent him from seeing its immediate bearings on practical medicine. But, while admitting that normal or physiological anatomy, which embraces in its limits both descriptive and general anatomy, furnishes the standard, deviations from which come within the domain of morbid or pathological anatomy, it does not follow that there should be a positive and unmistakable line of demarcation between the two. The regular and healthy structure of organized bodies often merges imperceptibly and in many different ways into that which is irregular and diseased. It has been well said that the idea of normal life is extremely indefinite, and it is only by a forced abstraction that the normal can be separated from the abnormal. We state this preliminary difficulty not with a view of discouraging inquiry, but rather of urging the necessity of a thorough investigation in both directions,—the healthy and the morbid structure,—so that the observer, by a familiarity with his subject, may be able to declare what is debateable ground, and what is clearly a deviation from the healthy or normal condition of the tissues and organs. Morgagni, than whom no one can be regarded as more competent to direct us in the premises, insists on a knowledge of both human and comparative anatomy as essential in the study of pathological anatomy.

Very crude and restricted notions of our branch are entertained by those who see in it nothing but an observation and record of the grosser changes presented by an organ or organs after death, such as redness, ulceration, morbid growth or attenuation, increased hardness or softness of texture, as regards the solids; and, as relates to the fluids, their effusion into some serous cavity, or on a mucous surface. We must not be supposed to underrate all or any one of these deviations from the normal anatomical condition of the organism; but we must go further, and, not content with noting the last stage of vital organic changes, apply ourselves to each individually, through its minutest details, and set forth its causes, gradual development, and consequences. To be able to do this implies the necessity of frequent *post-mortem* examinations, so that we may ascertain the changes of structure which have taken place at each successive stage of morbid development or disintegration, and appreciate the precise part performed by each tissue and organic system on the occasion. Hence the necessity of studying not merely the coarser changes, such as are visible to the naked eye, but also those finer modifications affecting elementary tissue, visible only by the microscope; in other words, of studying histology, or textural development. As histology is now a recognized integral part of physiological anatomy, so must it be regarded as equally essential to pathological anatomy; and, in like manner, as animal chemistry is a necessary ally to physiological anatomy and to physiology, so, of equal necessity, is its aid to pathological anatomy. It is the same science working very nearly by the same means in both branches; receiving in the first connection the title of physiological, and in the second that of pathological chemistry. This last is becoming a separate branch of science, but not as it seems to us beneficially for medicine, since we can hardly conceive of it as separated from pathological anatomy, of which it is a necessary complement if not an integral part. Enlightened by animal chemistry, we detect not only the differences between the secreted fluids, the product of healthy or physiological function, and those resulting from morbid changes in texture and functions, but also the changes in the blood itself, from which the other fluids are formed. In what are called general diseases this investigation assumes the highest importance, since, in the absence of local lesions, we must look to the blood alone for the material seat and cause of the disease, as in most fevers, for example. During the reign of the humoral pathology, when the blood and the various secretions were made to figure so conspicuously in the changes which they were believed to undergo, an explanation of the chief symptoms of disease was supposed to be furnished in this way. Disease was regarded as a struggle between these morbid and disturbing causes on the one hand, and nature on the other, who, exerting herself in opposition, attenuates, elaborates, overcomes, and expels them by the natural channels, when the case terminates favorably; or the disease is directed on organs essential to life, and nature is exhausted in vain efforts, when the case ends fatally. This might be called the period of the *pathological anatomy of the fluids.* Almost forgotten, except as belonging to the history of medicine, and ridiculed under the influence of solidism, which was inaugurated in modern times by Hoffman and Cullen, humoralism comes up, once more, in our own day, and takes a permanent place in the pathology of the schools, under not only a modified phraseology, but with the support of the microscope and animal chemistry; such as that exhibited, in relation to the latter, by Andral and Gavarret, in their *Hæmatologie:* and, once again, we can speak of a revival of the age of the pathological anatomy of the fluids.

Allusion has been made to the difficulty, in some cases, of distinguishing healthy from diseased structure; and hence an occasional cause of fallacy in *post-mortem* examinations. We cannot convey a better idea of the risk of mistakes of this nature than by repeating the illustrative remarks of Wedl on the subject:—" The excessive size or minuteness of an elementary organ, (cell,) its altered relative position, its changed contents, the various conditions of the *nucleus,* &c., are points demanding assiduous and strenuous investigation. In order to render the matter clearer, we will here adduce some instances:—The presence of fat in the hepatic cells is connected, to a certain extent, with the conditions of nutrition; and we should be very wrong, where the fat is present in small quantity, and without any other complication, at once in assuming the existence of a fatty degeneration of the cells. The same holds good with the fatty contents of the epithelial cells of the kidney, in which also the presence of fat depends, to a certain extent, upon their nutritive condition, and by itself alone, in a minor degree, should not be regarded as pathological, or in fact as at all sufficient to constitute a 'Bright's kidney.' The retrograde or atrophic modifications also of the tissues, occurring in old age, have not the same significance in a pathological point of view as they would have in younger individuals. A certain amount of atheromatous deposit in the vessels of a person eighty years old would necessarily be referred to the involution proper to the physiological condition at that period of life; while the same degree of the affection in the vessels of a youth of twenty would indicate a partial premature retrogression of the organism, and could only under such circumstances be of pathological interest. Hence it is evident, that, in the exposition of the abnormal condition of a tissue due regard must be paid to all the circumstances, and the more so in proportion as the anomalous state is less determinate." In the *post-mortem* examinations most frequently made, viz. of the digestive mucous membrane, how necessary do we find it to protect ourselves against erroneous deductions from hasty observations of the colors of the mucous surface; and from assuming as a morbid condition, a redness of the part, for example, that which is physiological at a certain age, and, as regards the individual, in certain periods of the digestive process.

In the study of pathological anatomy opinions must never be substituted for facts, as is too often done in careless examinations of the dead body. How common for us to be told that an organ is inflamed or bears marks of its having been inflamed during life, although no specification is made of the characteristics of this state; and yet a discrimination between it and other preternatural conditions is not always an easy matter. A frequent cause of fallacious induction consists, as Cruveilhier very clearly points out, in ignorance or oversight of the physical and chemical changes wrought in the texture of the organs, in the last agony, or immediately after death. The changes of most common occurrence are in color and consistence; and in them there is great variety. They are those which incipient putrefaction most readily develops. The vital principle withdrawn, or vital action ceasing, the relations previously existing between the solids and fluids are no longer preserved, and parts which during life gave passage to the fluids without combining with them, become after death like simple porous bodies and allow of free infiltration. The coats of the vessels, in the first instance, then the adjacent tissues, are colored as if by a process of dyeing; and hence vascular redness, to which so much importance has been attached, and hence, also, the redness of the tissues, regarded by some as evidence of great hyperæmia if not of actual inflammation. Passive sanguineous congestions, for instance, are often mistaken for the effects of inflammation, as in the accumulation of blood and serosity in the posterior portion of the lungs, which is partly a cadaveric phenomenon. A sanguineous stasis may be made in any part by rendering this latter dependent, whether it be the head or the feet. Softening of the tissue is an effect of this diffusion of the fluids beyond their customary vascular or cellular limits; and sometimes a finger may be thrust into the substance of the lungs or the liver, which has been softened by cadaveric infiltration, in the same way as if it were thrust into these organs when in a state of inflammation. So, also, of other fluids besides the blood. Scarcely can a body be opened without our seeing the transudation of bile through the sides of the gall-bladder, and, not unfrequently, even the contiguous intestine and parietes of the abdomen. Cruveilhier relates his having opened the bodies of persons who had died soon after a full meal, in which the gastric mucous membrane was dyed of a vinous tint by the coloring matter of the wine previously drunk. In other cases, the coloring matter had become blackish in the small intestine, which was of the same color. This author asserts that the phenomena of putrefaction are soon added to those of a physical nature just noticed; and that the greater number of the bodies opened in the hospitals must be regarded as in a state of incipient putrefaction. Beclard shows how the evolution of gas in the abdomen, by throwing up the diaphragm compresses the abdominal vessels, causes a reflux of blood to the heart and the veins of the upper part of the body; and hence injection of the face, redness of the eyes, contraction of the pupil, and sometimes hemorrhages, if we can designate by this term cadaveric transudations. Whenever we meet with diffused redness, without arborescent or tufted vascular injection, we should regard it as a physical phenomenon. Of the same nature in many cases is the softening of the tissues noticed in *post-mortem* examinations.

Pathological anatomy is declared to be a modern science; certain it is that only of late years has it assumed the dignity of an independent one. The irregular and slight contributions made to it, in ancient times, by the school of Alexandria interest us more as belonging to the literature of medicine than as forming an essential part in the progress of the branch now under notice. Outlines of its history are given by Cruveilhier, Otto, and Rokitansky, to whose volumes we refer our readers for information on this point, which we do not deem needful to introduce here. It is enough for us to mark the chief eras, and name the distinguished few who have contributed to create them. At the beginning of the sixteenth century, the period of the regeneration of anatomy, we may date the rise of "an occasional, fragmentary, indeterminate study of pathological anatomy." The importance of this latter, however, had been early felt, as Eustachius, the rival of Vesalius, toward the close of his life "expresses his regret that he had not rather bestowed upon pathological anatomy that time and attention which he had devoted to physiological anatomy." To Italy, which took the lead in the restoration of anatomy, are we indebted, in the person of Antonio Benivieni, for the first work on the subject, entitled, *De Abditis Morborum Causis*, written in 1507. Rokitansky, after enumerating other laborers in this line—including Bonetus or Bonet, who was the first to compile an ample repertory on the subject, (*Sepulchretum*, 1679,) and Blankaard, (*Anatomia Practica*, 1688,)—passes the following animated and expressive encomium on Morgagni, who wrote nearly a century later :—

"Above both these—above all that had been previously accomplished—stand pre-eminent Morgagni and his work *De Sedibus et Causis Morborum*, (1767.) Notwithstanding its defects, this book remains a model of industry and perseverance, of method and arrangement, of breadth and perspicuity, and, lastly, of originality, for all time."

The opening of the present century was made ever memorable by the genius of Bichat, who in addition to his great merits as the founder of general anatomy, and his being the first to make the attempt "to treat histology scientifically," gave the most decided impulse "to a right conception and application of pathological anatomy."* Pathologists imitating him, endeavored to reconstruct their science upon an anatomical basis. France was the land, of all others, in which men sought and found in pathological anatomy a solid foundation for medical knowledge. "Such men were, among others," continues Rokitansky, "Bayle, Corvisart, Laennec, Dupuytren, Broussais, Cruveilhier, Rochoux, Lallemand, Riobe, Andral, Louis, Gendrin, Bouillaud, Billard, Rayer." "In England many have, up to our own day, worked in a similar spirit. Among these we may mention the names of Abernethy, Charles Bell, Astley Cooper, Hodgson, Farre, Wardrop, Howship, Baron, Hodgkin, Hope." The list might be extended; and in no case ought the names of Abercrombie and Stokes to be omitted. "In Italy, on the contrary, and in Germany—if we except the impulse given in the same direction by the ingenious Reil—pathological anatomy has been, upon the whole, less cultivated, and has exercised less influence upon medicine. Accordingly, Germany and Italy have but few men to place in parallel with those of France; few to add to the names of Scarpa, Malacarne, Paletta—of J. F. Meckel, Otto, and (in industry and method the essentially German) Lobstein." The future historian of pathological anatomy will not fail to rank the name of Rokitansky himself on the first line of German laborers in this field; and after him, but at not a long interval, some of his eminent contemporaries, who, be it said, were entitled to a place in his own record, brief as it is.

The name of Bichat reminds us of the first of the aids to other branches of medicine imparted by pathological anatomy, viz., that to general anatomy. This eminent man, whose mind was both suggestive and experi-

---

* We do not take any account of a posthumous volume, made up of imperfect and not well-authenticated notes of Bichat's lectures, issued under the auspices of Beclard, with the title of *Anatomie Pathologique*. This work is not received as any authority by the French medical critics.

mental, was not content with testing carefully the normal, physical, and vital properties of a healthy texture, or a membrane, in order to ascertain its affinities and to determine in what class it should be placed, but he had recourse, also, to an observation of the morbid changes in its structure and products, either in the human body or as the results of experiments for the purpose on animals. In this way, even with more certainty than by resemblances offered in their healthy state, he was enabled to classify, under one common head, membranes in different and remote regions of the body and forming a part of quite different organs; as he did with the serous membranes, for example.

Physiology, also, is indebted to pathological anatomy, as when we learn the exact relation of function to organ, in the excitement, disturbance, or abolition of the former, consequent on the irritation, injury, or destruction of the latter; a relation better determined in this way than by vivisections of animals. In the first case, after a subsidence of the inflammatory stage, and when the lesion has assumed a subacute or more definitively chronic character, the line of demarcation is drawn that separated the lost or atrophied part from the remaining healthy one; and the functional differences then give the exact measure of the structural disorganization or loss—a fact this which no mere study of symptoms alone could have revealed to us, but for the observation of these in connection with a knowledge of morbid structure obtained after death. In the case of vivisections, it is difficult to restrict the experiment within the precise limits of the part or organ, so that contiguous textures shall not also be implicated; and besides, the sympathetic disturbances are for a while so great, and throughout the whole period of the experiment there is such a degree of irritative excitement, that the disorder or the loss of function which should result merely from the injured organ is masked and complicated. The destruction of an organ, as revealed by pathological anatomy, takes time for its completion, and at last ceases to awaken any very disturbing febrile and constitutional derangements. Hence the loss is analogous to congenital defects, as in monstrosities, and enables us, as we would in the latter, to say not only what are the functions withheld from exercise by the loss of an organ, but also the degree in which the latter is necessary to the vitality of the entire organism, and how far its structure is connected with that of other organs. We might adduce vivisections of the brain and nerves of animals, anatomico-pathological lesions of these parts in the human body, and anencephalous fœtuses, in illustration of these remarks.

Pathological anatomy may be said to be an integral part of clinical medicine, constituting as it does the chief basis of diagnosis, without which there can be no certain indications for the treatment of disease. Here we must remind our readers that we have claimed for our branch more than its being the mere exposition of the last lesions of tissues or of an organ which precede death. Thus restricted, it would possess comparatively little value for practical medicine. But when we contemplate morbid anatomy as making us acquainted with the successive stages of disease, resulting from organic processes, which it scrutinizes and explains, it assumes a higher purpose and more extended attributes. If we admit that symptoms are but the expression of a suffering organ—the effects of an antecedent cause, it is to this latter that we must direct our chief attention, and to it address our remedies. Some have, indeed, imagined a disease to consist in a group of symptoms, which are made to represent an abstract idea, in place of positive material derangements of the living organism; and in this sense these persons set themselves about combating an imaginary entity as they would a train of symptoms, the supposed effect of demoniac possession. The therapeutics based on such a pathology are, of course, purely tentative and uncertain, and at the best tedious, even if by chance successful. Each symptom is met by the administration of a particular medicament, which is supposed to possess properties capable of its removal; and thus the chase is continued in detail, and valuable if not irrevocable time is lost while the mischief of internal organic lesion is going on. The most violent symptoms—those which are most distressing to the patient, and often alarming to the practitioner, who rests his nosology on mere symptomatology—do not represent or leave behind them any serious lesion: they are but remote or secondary effects of an internal organic cause. Thus, for example, the burning heat of the skin, the thirst, the continued restlessness, the pain in the muscles and sometimes even in the bones, lassitude and extreme weakness, common in fevers and some of the phlegmasiæ, give no clue to the kind of fever or measure of its intensity: they are not the immediate results of morbid organic action or changes in these parts. If we add discoloration of the skin, as when it assumes a jaundiced or yellow hue, we cannot tell from this symptom whether the case is one of bilious or of yellow fever, or whether the stomach, or the liver, or the duodenum is the organ chiefly suffering. The patient sinks exhausted by the violence, sometimes it would seem by the duration, of the disease; and reference being had to the symptoms or external evidences alone, it might be said, that in the first stage he had been burned up, consumed by the excessive heat and thirst of which he continually complained; and in the second, that he died from inanition, caused by his inability to take and to digest food. All is doubt, uncertainty, and mystery! If sounder views of disease are entertained, if facts have been substituted for conjecture, and a careful analysis made of the phenomena of disease, and inductions only admitted from material and recognizable premises, we must seek for the cause of this reform and amended philosophy of medicine in pathological anatomy. The improvement in the one has kept pace steadily with the advances made in the cultivation of the other. Inspection of the body after death shows that the prominent symptoms, those of which the patient incessantly complained, were of minor moment, as far as regards their being indicative of the progress and termination of the disease; and that those less noticed, and regarded as merely epiphenomena, were truly the expression of the suffering and altered organ, which had been obscured and overpowered by the sympathetic disturbances of other organs. The true value of a symptom can only be measured by the condition of the organ in whose functional exercise it is developed. At times, we are able, by means of *post-mortem* examinations, to go beyond what in life seemed to be the co-relation of symptom and organ, and thus to gain a lesson of caution for our subsequent guidance. In the patient before us we may think we have to deal with a case of original asthma: it turns out to be an organic disease of the heart. In another we are treating hepatitis, when it is in fact pneumonia.

All that we know of physical diagnosis, as it is termed, is based upon pathological anatomy, which teaches the relation between certain sounds elicited by percussion and auscultation, and the morbid alterations of structure and secretion in the pulmonary and cardiac organs. Wanting a correct appreciation of the one, the other would be as unmeaning in diagnosis as the murmuring of an Æolian harp or sounds caused by the vibrations of musical glasses. It was not by a series of lucky guesses or plausible analogies that Laennec and Piorry were able to lay down those rules of physical diagnosis, and the connection between it and vital diagnosis, which now guide us in our daily practice, and place us in advance of the observation of symptoms alone, though prosecuted for many centuries. To what but to morbid anatomy are we to ascribe the different construction put on hemoptysis, both in regard to its diagnosis and prognosis, and also its treatment, at the present time, from that in all preceding ages? Regarded as a simple hemorrhage connected with more or less general plethora or pulmonary congestion, it was treated accordingly; and of course, often by free venesection and antiphlogistics generally. Its relation to phthisis was believed to be, if not causative, at least as a precursor to the latter. But now, thanks to pathological anatomy, we have learned that, in a majority of the cases of hemoptysis, the hemorrhage is an effect and one of the symptoms of a tuberculous lung; and as such we are chary in abstracting blood, and in the use of reducing treatment, beyond what are urgently demanded by the associated pulmonary congestion; knowing that a reducing course is contraindicated in tuberculous disease. Our suspicion of this sinister connection of hemoptysis is greatly strengthened by our detecting, by means of percussion and auscultation, an alteration in the pulmonary tissue in the upper portion of the lung, in which pathological anatomy has taught us that tuberculosis begins.

The entire pathology of phthisis, both in relation to cause, inception, progress, and termination, and the modified views of the effects of remedies, at the present day, are almost entirely due to pathological anatomy, which, if it has not pointed out a successful treatment, saves us, at least, from much empiricism and false doctrines, which would prompt to a resort to useless and damaging therapeutics. It has also shed a ray of hope for the consumptive invalid, and offers encouragement to the physician, by showing that consumption has been cured by an arrest of the morbid processes which were going on in the lungs, and, at times, by the conversion of tubercle into calcareous deposit; although we are still ignorant (despite the assurances to the contrary of lying empirics in and out of the profession) of any therapeutic or other means by which these salutary changes can be brought about.

Cardiac pathology, lost or obscured in what was believed to be other separate diseases, such as asthma, dyspnœa, and palpitations, has, since the time of Corvisart, half a century ago, attained to a precision in its diagnosis and a knowledge of its structural alterations and their effects, for the like of which, if we except the contributions of Morgagni (Letters 17 and 18) in his great work *De Sedibus*, and of Senac, as contained in his volumes on the structure, action, and diseases of the heart, we look in vain to all that had been done in this line during the long preceding period of more than two thousand years. This wonderful progress and its results are the fruits of pathological anatomy, which has imparted an intimate knowledge of the changes in the nutritive life of the muscular tissue of the organ, (hypertrophy and attenuation, softening and fatty degeneration of its walls,) of inflammation of its lining and investing membranes, (endocarditis and pericarditis,) and of constriction, patency, or ossification of its valves. From the same source we have derived an acquaintance with the relations between diseased heart and diseased brain, as perceived when hypertrophy of the left ventricle is associated with apoplexy. So, also, in dilatation and weakened powers of contraction of the right side of the heart, and in valvular obstructions, both of which prevent a free and ready return of blood by the vena cava, and induce consequent retention of this fluid in the veins generally, we find the chief cause of passive dropsy.

A yet more recent triumph of pathological anatomy is exhibited in the discovery of that morbid or granular structure of the kidneys, which, by disordering their functions, brings with it a number of other diseases—each one of them formerly treated as primary or of distinct occurrence; or if, as in the case of dropsy, it was regarded as secondary, a wrong cause was assigned for its appearance.

The diseases of the nervous system are commonly represented to be less indebted for the elucidation of their pathology by the detection of a material and structural seat, or accompanying organic lesions, than affections of other parts of the organism. In the main this belief is well founded, but even here the domain of semeiology, if not of therapeutics, has been greatly extended by a careful application of pathological anatomy to our investigations in this department. We may cite, in proof, the familiar example of apoplexy, in which the extravasation of blood and deposited clot in the brain affords an explanation of certain symptoms, such as paralysis and anæsthesia; while, at the same time, it teaches us the futility of attempting, by any class of remedies, to bring about a speedy absorption and removal of the effusion, and consequently suggests the necessity of our admitting time as an important element in the treatment of the case. That other lesion of the brain termed softening, which is followed by distinctive affections of the mind and muscular system, is one of the modern discoveries effected by morbid anatomy. Here, still less than after apopletic seizure, can we hope, with a knowledge of organic cause, to bring about a restoration of healthy structure and a removal of the disease; but we are able to display the negative merit of withholding a number of wearying, if not injurious trials of various therapeutic agents, which practitioners, when less enlightened on the subject, deemed it to be their duty to make.

A knowledge of the successive stages of the lesions of internal organs constituting inflammation, and the corresponding diseases of function, can only reach us through morbid anatomy. The entire value of the pathognomonic symptoms of pneumonia in establishing a diagnosis, and to a great degree a prognosis, and in regulating the treatment, depends on their representing the changes of congestion or infiltration, hepatization and suppuration, which the inflamed lung undergoes in the progress of the disease. Aware as we are that in inflammation of a viscus, as indeed in all the phlegmasiæ, a certain period must elapse from its inception to its termination, we can easily estimate at their real worth the pretensions of those who claim the ability to arrest the disease, or to cure it in an incredibly short time. The separation of croup from other diseases of the respiratory passages is due to pathological anatomy; and under the same guidance have we been enabled to divide this disease into—1st, the simple catarrhal and spasmodic; and 2d, the membranous. In our fear of the latter, we are led to the adoption of an energetic treatment of which the collective symptoms might not always indicate the necessity. By the same means also a diagnosis is made to establish the distinction between croup proper and certain anginose affections marked by membranous exudations on the mucous lining of the fauces and pharynx as well as of the larynx; and on this distinction, added to our knowledge of their external causes, is based an essential difference in the treatment of the two diseases.

We have now adduced proof enough to make out our case; and to show, beyond question, the great and beneficial applications of pathological anatomy to the different branches of medical science, including in a more especial manner clinical medicine. We might, if we were writing an essay on the subject, increase and diversify the illustrative proofs of this truth. Surgery, without pathological anatomy, would be reduced to a mere mechanical art, aided by certain empirical practices, alike unsatisfactory to the surgeon and of doubtful benefit to the patient. The union of divided and the restoration of lost parts of the animal organism, the genesis of morbid growths and products, and their differences in structure and progress, as, also, in their readiness to call into sympathetic disturbance the different functions, and to bring on disease and death, would all be subjects of conjecture and mystery to the surgeon who is unenlightened by pathological anatomy. When to amputate a limb or to excise a tumor—what will be the probable consequences of the operation—what the constitutional treatment necessary to prevent the patient from being carried off by consecutive inflammation and fever, or from sinking at once under the shock of the operation—are questions which the unenlightened surgeon will be at a loss to answer, if, indeed, he has the perspicacity to present them at all to his own mind for impartial adjudication.

Medical jurisprudence rests largely on pathological anatomy, as in the investigation of all cases of homicide and suicide—whether they be the result of wounds, suffocation, and strangulation, or of poisons, even although in this last case the chemical tests are all-important. The question of cadaveric, as distinguished from pathological alterations of tissue, and the different effects of putrefaction after the body has been some time submerged in water or buried in the usual way, belong to the same category; as do also those of abortion and recent childbirth, when the expert is required to ascertain whether such processes have been gone through. A more precise knowledge of morbid anatomy, in its various applications to medical jurisprudence, possessed by the members of the medical profession generally, would furnish aid essential to justice, and, at the same time, prevent the mortifying exhibition of contradictory opinions when they are summoned to give evidence in a court of law. As things now are, every sprig of law, after a day or two of cramming by his looking into a work on medical jurisprudence, has it in his power to embarrass, perhaps to stultify, the medical witness in the case, who, if ignorant, will be ashamed to avow it, and will be apt to indulge in all kinds of random conjecture, and to go on from one blunder to another, until court, jury, and audience are convinced of his incapacity, and, worse still, draw the erroneous inference that medical science is not capable of throwing light on the subject. When will our schools be found imitating the medical jurists of Germany, who designate pathological anatomy applied to medical jurisprudence by the title of forensic anatomy, (*anatomia forensis*)?

Great as are our obligations to pathological anatomy in the investigation of acute diseases, they are greater still in that of chronic ones. The affected organ in the latter state of things speaks to us by fewer and less active sympathetic disturbances, and hence it is the more necessary to sift and examine the evidence furnished by the symptoms; but its own lesions are on this very account less masked by a crowd of these symptoms. The slow fever, the gradual wasting away of the frame, and loss of strength and spirits, alternating sometimes with fitful gleams of cheerfulness, afford unmistakable proofs of disease, and of disease kept up by an organic cause. There is a thorn in the flesh, the nature and progress of which are revealed by pathological anatomy, and by this revelation we are put in the right direction of cure, without, however, the precise means being indicated. We learn the slowness of the morbid changes of structure in the organ, and also the length of time for the recuperative ones to be gone through before we can hope for the desired restoration to health. We have recourse, in consequence, to a plan of treatment which shall effect our object without violence to the system, and aid nature, in the processes which he sets up, viz. the absorption and removal of morbid deposit in the organ, and the renewal of healthy secretion and of nutrition. The great element of time having been required by the physician and conceded by the patient, to whom a plain and untechnical explanation of his state should be made, the entire plan will then be developed. It will consist of an alterative course in the largest sense of the term, and will include not only recourse to medicines of this class, but also to the chief agents of hygiene, and the means, if possible, of agreeable mental occupation.

Our cause on the present occasion is too strong and is fortified by too great an array of facts, presented in too many different shapes, to justify special pleading or to allow of our leaving unnoticed some objections which may seem to weaken the force of the general argument. When we said, a while ago, that symptoms are but the expression of a suffering organ, it might have been replied, that, granting the fact, it resolves itself into an abstract proposition, of the truth of which we sometimes find no material proof in alteration of texture of the organ thus suffering. Apoplexy sometimes kills without any perceptible rupture of vessels, serous effusion, or other lesion of the brain; the same absence of lesion marks some cases of epilepsy; and we have ourselves seen convulsions followed by coma, strabismus, and other symptoms of acute hydrocephalus, or, as it would now be called, meningitis, and death without encephalic or meningeal alterations. It is not an uncommon thing for us to be baffled in our search after structural changes in subjects who have died of fevers of different types. Many years ago we lost an estimable youth in declared and satisfactory convalescence, after an attack of fever resembling in its classification a mild case of typhoid: the death was sudden and unexpected. A *post-mortem* examination made for us by the accurate Horner failed to reveal, either to him or to other parties present at the time, an appreciable lesion of any of the great organs or membranous systems. Asthma, for the most part dependent on or symptomatic of cardiac or of pulmonary lesion, is sometimes purely nervous; or, at least, it is not associated with any noticeable deviation from the healthy structure of the organ which is its seat. Most of the diseases which we call organic find their homologues, so far as symptomatology is concerned, in affections entirely nervous. In some diseases the structural lesions, if they existed at all, disappear just before or at death. And, again, when we meet with extensive structural alterations of a viscus or viscera after death, from certain kinds of fever, as of the stomach in yellow, of the liver and duodenum in bilious fever, and of the lower end of the small intestines and the spleen in typhoid fever, how should we speak of them? As primary lesions, or secondarily sustaining causes of the fever?

Granting the full weight which may be attached to these objections, they only go to show, what no thoughtful person can deny, that pathological anatomy is far from being a complete science, and that the difficulties enumerated are only proofs of the necessity of a continued and thorough inquiry, with the aid of the microscope and of chemistry, into pathological histology and anatomy. The occurrence of functional disorder, and serious and even fatal disease, the direct effect of structural alterations and lesions cognizable by our senses, will not allow us to doubt, for a moment, that the like effects will ensue from internal local causes. Apoplexy and palsy, such as we commonly meet with, are not the less produced by extravasation of blood from ruptured vessels, because under rare and as yet unknown circumstances these diseases may occur without perceptible lesions; nor is it less evident that in the former case the restoration of sensibility and mobility will take place in the ratio of the removal by absorption of the blood-clot or of the serum, which, by its pressure on the cerebral organ, had given rise to the abolition of intellect and of sensorial and motor power. It may be made a question, in a lack of full details of the cases, whether the sudden deaths attributed to dry apoplexy were not really caused by organic lesions (valvular obstructions, or the like) of the heart. In reference to the belief that phlogosis and engorgement of one or more of the viscera may be an effect instead of a cause of febrile disease, we need not engage in a dispute about the order of succession. It is sufficient for us to learn from pathological anatomy that the whole force of the disease is spent on a particular viscus, in order to induce us to take speedy and active measures for either carrying off a first cause of admitted mischief, or of mitigating the violence of an effect which is a secondary cause, and which may be equally injurious as the first.

It is justly said by Vogel that pathological anatomy derives the materials of its knowledge from two separate sources. First, from *observation*, which includes the examinations of portions of the living body altered by disease, and removed either by the knife of the surgeon or thrown off as excreta, (in the widest sense of the word,) together with the examination of the dead body, which is often able of itself alone to explain to the practiced observer the whole course of the disease. In most cases, continues this writer, it is, however, of importance that the examination of the dead body should be supported and perfected by observations made during the progress of the disease. It is also essentially important to therapeutics that pathological anatomy should be pursued by scientific, unbiassed pathologists, rather than by mere anatomists; as we must be very careful in tracing the connection which subsists between the symptoms during life and the changes noticed after death.

The other mode pointed out by Vogel, by which pathological anatomy gains new facts, is by *experiment*. Experiments instituted on animals, and, in certain cases, on man, with the view of artificially producing a morbid condition, and then carefully studying it, afford most valuable aid; for here, as he alleges, the quality and quantity of the causes in action are much more under control than in ordinary cases of disease. On this point our own opinion, already given, is somewhat different from that of the German professor.

Wedl, in reference to the means by which pathological observations are to be carried on and histological researches made, tells us:—"The *methods* followed in pathologico-histological researches may be described, as it were, as *synthetic* and *analytic*. The latter commences with the elementary constituents, and proceeds to inquire into their disposition and relation to other elementary parts, their various chemical properties, their diversities in different parts, &c. In the *synthetic* method, the secondary arrangements of the elementary parts grouped together, are investigated. For the *analysis*, the most powerful object-glasses of the compound microscope are employed; for the *synthesis*, the lower powers and the simple lens. The course to be pursued in any particular case depends upon the plan of investigation.

"The *simple lens*, or microscope, is an indispensably requisite instrument, and every observer should take care to be provided with good lenses of this sort, with which alone many observations in their totality can be undertaken; as, for instance, the distribution of the larger blood-vessels, their course and relation to various parts of a tumor, or the areolated tissue of a cystic thyroid gland, &c.

"It is equally necessary in the preparation of more delicate objects. The powers available should vary from four to fifteen diameters. The lenses should be supported on a firm stand, so arranged as to allow of the object being viewed in various positions, and, if need be, by transmitted light. A watchmaker's lens is recommended by some observers.

"Researches by means of the *compound* microscope involve a tedious, though absolutely necessary, detailed investigation of the component parts of the diseased tissue, already seen to differ by the naked eye. The microscopical examination, therefore, starts from what has been seen by the naked eye, and presupposes a strict observation by the latter; and it is evident that pathologico-histological research of this kind must demand considerable time."

The requisite aids in researches of this kind, as enumerated by the author, are *distilled water; acetic acid,* for the display of *nuclei; sulphuric acid,* as a useful reagent, for instance, to detect the presence of carbonate of lime in concretions; *hydrochloric acid,* occasionally useful in the investigation of morbid structural changes in the bones and teeth; *nitric acid,* much diluted, may be employed under the microscope to show the change in color of the coloring-matter of the bile, for giving a yellow color to organic muscular fibre, the hardening of organs, &c.; *chromic acid,* much diluted, [or a solution of bichromate of potass,] may be employed for the hardening of morbid tissues in order that sections may be made; *caustic potass and soda,* or *the carbonates of those alkalies* are extensively useful for the display of elastic tissue of the nerve-fibres, or rather of the medullary matter contained in them, for giving transparency to connective tissue and to muscles; *solutions of common salt and of sugar* (a few grains to the ounce of distilled water, so as to make a solution in which the blood-corpuscles do not contract) are employed in tracing the distribution of the blood-vessels; *plain water,* for depriving the blood-corpuscles of their coloring-matter. In the case also of very delicate or easily-ruptured cells, it is advisable to employ one of those solutions, since they are injured by the imbibition of plain water. *Goadby's fluid,* composed of four ounces of salt, two ounces of alum, four grains of corrosive sublimate, dissolved in two quarts of boiling water, may be employed either as a preservative agent or for the purpose of hardening tissues; *alcohol* is very valuable as a preservative agent, and also for the purpose of hardening tissues. Thin sections, also, of preparations made with alcohol, and treated with acetic acid or carbonate of potass, are very instructive in many respects. *Tincture of iodine* is useful, partly for the coloring of objects, partly on account of its reaction on amylaceous granules; as, for instance, in fœcal matters and upon fungi.

"The *instruments* necessary for the pursuit of histology are few and simple. They consist of common needles and straight cataract needles, some straight and fine-curved scissors, hooks, scalpels, razors, fine forceps, slips of glass, pieces of thin glass or talc for the covering of objects, fine glass tubes, a metacarpal saw with moveable blades, having teeth of different degrees of fineness, several files, probes, and hair-pencils, slices of corks," &c.

If we look at pathological anatomy in an exoterical point of view, and inquire into the position which it holds in our programmes of medical education, we see little else than an entire neglect or depreciation of its worth. Principles are every now and then dwelt on, while that which underlies all principles is overlooked: cautions are uttered, in solemn oracular style, against theories and speculations and hasty judgments, while the only certain check and corrective—inductions drawn from carefully-observed phenomena of organized matter—is concealed and too often contemned. A well-educated physician coming among us, as a stranger, but who had heard of our numerous medical colleges, might very naturally be supposed to express his satisfaction at the prospect thus offered for a thorough inculcation of this subject in all its bearings, and by competent teachers, on the thousands of eager youths who resort every winter to our schools. What will be his surprise when he reads the following sentence in the preface to Dr. Gross's *Elements of Pathological Anatomy:*—"In the forty-five medical colleges which at this moment exist in the United States, there is not, with perhaps a few exceptions, a chair of pathological anatomy: thus clearly showing that the teachers connected with these institutions have either no very exalted opinion of the value of this science, or that they are afraid of innovation." As a separate branch it very seldom forms a part of the curricula of our medical schools; and when it is announced to be taught in connection with another branch, or sometimes other branches, from one chair, there is no security for its really becoming a definite and appreciable part of the prelections and demonstrations of the professor. It is like a ball tossed from one teacher to another, and caught or let drop as suits the fancy of the hour. Sometimes it is introduced into a school as the subject of a separate chair, not so much from a conviction of its intrinsic importance as from a desire on the part of the Faculty to secure the support of a person of some influence, and who must, it is alleged, be provided for. His qualifications for the post are not very rigidly inquired into; and whether this be owing to the indifference of his new colleagues to the subject and their ignorance of its real character, or their good nature, we need not say. The practical commentary on the whole transaction is offered in the fact of the new incumbent at the next reorganization of the faculty, or after the death of a brother-professor, abandoning his chair, which is in consequence abolished; and he takes one of the old-fashioned series, the duties of which are more congenial to his tastes and do not tax him with any additional effort of thought or attainment.

In looking over the announcements of the courses of instruction for the present season in twenty-one medical colleges, which include the leading ones of the United States, we do not find a distinct chair of pathological anatomy in a single one of them; nor is it named at all even in connection with other branches in more than three of the entire number, and then it is with and coming after physiology. We look in vain for a chair on the subject in either of the schools in Cincinnati and Louisville, in the former of which it was once taught by Dr. Gross, and in the latter by his eminent friend Dr. Drake. Its separate existence is ignored in the school with which Dr. Gross is now connected, as it is in the rival one of the University of Pennsylvania. The most that can be conceded to these two institutions in the matter is the chance good-luck of a professor in each of them being the author of a work on *Pathological Anatomy;* but as far as regards the fostering influence, the genial warmth of sympathizing associates in either of them, we are afraid that these productions would have seen the light as readily on the other side of the Atlantic as here. The creditable *Treatise* by Dr. Horner has not, after an interval of more than a quarter of a century, as yet produced public fruits nor secured a continuance, on the part of others, in the course which he began. Whether Dr. Gross's *Treatise,* rich as it is in positive knowledge and suggestive reasoning, will be productive of happier results, remains to be seen. We are glad to be able to mention one exception to this general neglect. It is in the instance of the medical department of Harvard University, in which pathological anatomy is so worthily taught by Dr. J. B. S. Jackson.

We shall be told of the difficulty of multiplying the demands on the time and attention of a student whose mind is already greatly tasked, and must necessarily be still more so by the addition of a new chair. Something also may be hinted about increasing the expenses, already considerable, of the young men who resort to the lecture-rooms of our medical colleges. But the primary and permanent consideration, in questions like this, is its bearing on medical science and medical art, and through these on the ability of medical men to do the greatest good in their power to the community. Now if it can be shown that pathological anatomy is an indispensable basis and support of both medical science and medical art, and that a competent knowledge of it will strengthen the hands of medical men in their professional ministrations, we are not justifiable in refusing to give it all due countenance, and to enforce its precepts from a fear of any difficulty either on the score of mental labor or increased expense. We shall encounter a more serious difficulty than all this in an attempt to reconcile to our convictions of right an economy of time and money at the cost of the lives of our fellow-creatures. Suppose us to be for the nonce, what in fact, however, we are not, of the number of those who assume or are appointed to the office of teaching medicine, we must not allow the acknowledged difficulties of our position to cloud our perception of responsibilities of a very momentous nature incurred from the moment of our installation. Professors ought not to subject themselves to the taunting remark, that an amount of thought and determination devoted to the recognition, advancement, and thorough teaching of the different branches of medicine, equal to that manifested in swelling the numbers of their classes and watching over the fiscal concerns of their schools, would obviate the objections and smooth the difficulties presented by the timid, the ill-informed, or the selfish.

There is yet another consideration which cannot be without weight in all questions of reform and progress in the age in which we live. It lies at the foundation of all our medical institutions, and concerns their very existence. The most superficial observer can hardly fail to notice the desire for change and the impatience of restraint by which the whole world is agitated at the present time. There is a spirit abroad which, although it may disclaim an intention to destroy, is so intent on reconstruction that it is anything but nice in the selection of its materials, and cares little whether these be new or are procured from old edifices. It exhibits itself both in opinions and in practices: sometimes in fitful and instinctive longing for improvement, but in ignorance of means and direction; sometimes in a more measured fashion, in which the discontent of the crowd is cunningly shaped by the designing and unscrupulous few. These disturbing elements set loose, the friends of social order, the conservatives of what they believe to be right, are called on to rally for the protection of themselves and institutions under and amidst which they live; but in order that they may do so with effect, they must be sure that they have both right and reason on their side; and that they do not become, without being conscious of the fact, the advocates of error because it is old, and the opponents of truth because it is brought before them without due preparation and recognized forms and precedents. The truly intelligent friends of medical science and medical institutions, much as they deprecate ignorant pretension and the vulgar clamor of empirics and their adherents, as well as the absurd speculations and practices which spring up to the wonderment of the multitude, are not on this account to be classed with those who, by their conduct if not by their language, assert that medical institutions have reached their final limits, that to press beyond these is desertion, and that reform is revolution and innovation destruction. The true friends of medicine are also friends of improvement, and, fortified by the uniform experience of history, they believe that the way to prevent destructive revolution is to institute timely and salutary reform; and that if ignorance and lawlessness should triumph, it will not be by their own strength, but by the unreasonable tenacity of purpose and waste of power and influence in protecting old abuses on the part of those who pass for wise. In the struggle, that particular point may be reached when moderate and good men, seeing that they are not able of themselves to bring about desired changes, owing to the steady opposition of those who are in possession of the high places and strongholds, will confine themselves to the station of passive spectators, and take no steps to prevent or to check the assaults of the excited multitude—the particolored revolutionists; but, on the contrary, they will finally bring themselves to prefer the dangers of subversion and the uncertainty of wise reconstruction to the continuance of abuses and obstructions which stand prominently in the way of political, social, or educational reform, as the case may be.

Let us hope that the regular medical institutions of our country will profit by lessons of this kind; and, at any rate, that they will not allow it to be said that he who aspires to be an accomplished or even a thoroughly educated physician must seek outside of their walls for the requisite aids and appliances which they do not possess, or will not use, inside of them. Pathological Anatomy, Medical Jurisprudence, Public and Private Hygiene, Medical Botany, and Medical Biography and Bibliography, are on the outside or excluded list. These are not subjects of mere dry lore or of speculative inquiry: they have a directly practical and appreciable bearing, as evinced in European schools, in which each is taught from a separate chair.

The recent establishment of a new Pathological Society in Philadelphia, and the existence of similar associations which are actively at work in Boston, New York, and Baltimore, show unmistakably the opinion of the more intelligent and advancing part of the profession on the subject in this country.

